# Implementation of video-calls between patients admitted to intensive care unit during the COVID-19 pandemic and their families: a pilot study of psychological effects

**DOI:** 10.1186/s44158-022-00067-2

**Published:** 2022-08-23

**Authors:** Filippo Sanfilippo, Luigi La Via, Giovanni Schembari, Francesco Tornitore, Gabriele Zuccaro, Alberto Morgana, Maria Rita Valenti, Francesco Oliveri, Federico Pappalardo, Marinella Astuto, RODOLICO-I. C. U. research group, Cesare Cassisi, Alfio Castro, Sergio Cocimano, Fabio Criscione, Carmela Cutuli, Veronica Dezio, Giuseppe Fallico, Monica Leonardi, Marta Mascari, Antonino Paratore, Francesco Perna, Milena Pulvirenti, Eleonora Tringali, Francesco Vasile, Maria Teresa Agnello, Giulia Sanfilippo, Simone Messina, Federica Merola

**Affiliations:** 1 Department of Anaesthesia and Intensive Care, A.O.U. “Policlinico-San Marco”, Catania, Italy; 2 School of Anaesthesia and Intensive Care, University “Magna Graecia”, Catanzaro, Italy; 3 Cardiothoracic and Vascular Anesthesia and Intensive Care, AO SS Antonio E Biagio E Cesare Arrigo, Alessandria, Italy

**Keywords:** Depression, Anxiety, Quality of life, ICU

## Abstract

**Background:**

The coronavirus disease 2019 (COVID-19) pandemic has caused over 530 million infections to date (June 2022), with a high percentage of intensive care unit (ICU) admissions. In this context, relatives have been restricted from visiting their loved ones admitted to hospital. This situation has led to an inevitable separation between patients and their families. Video communication could reduce the negative effects of such phenomenon, but the impact of this strategy on levels of anxiety, depression, and PTSD disorder in caregivers is not well-known.

**Methods:**

We conducted a prospective study (6 October 2020–18 February 2022) at the Policlinico University Hospital in Catania, including caregivers of both COVID-19 and non-COVID-19 ICU patients admitted during the second wave of the pandemic. Video-calls were implemented twice a week. Assessment of anxiety, depression, and PTSD was performed at 1-week distance (before the first, T1, and before the third, video-call, T2) using the following validated questionnaires: Impact of Event Scale (Revised IES-R), Center for Epidemiologic Studies Depression Scale (CES-D), and Hospital Anxiety and Depression Scale (HADS).

**Results:**

Twenty caregivers of 17 patients completed the study (T1 + T2). Eleven patients survived (*n* = 9/11 in the COVID-19 and *n* = 2/6 in the “non-COVID” group). The average results of the questionnaires completed by caregivers between T1 and T2 showed no significant difference in terms of CES-D (*T1* = 19.6 ± 10, *T2* = 22 ± 9.6; *p* = 0.17), HADS depression (*T1* = 9.5 ± 1.6, *T2* = 9 ± 3.9; *p* = 0.59), HADS anxiety (*T1* = 8.7 ± 2.4, *T2* = 8.4 ± 3.8; *p* = 0.67), and IES-R (*T1* = 20.9 ± 10.8, *T2* = 23.1 ± 12; *p* = 0.19). Similar nonsignificant results were observed in the two subgroups of caregivers (COVID-19 and “non-COVID”). However, at T1 and T2, caregivers of “non-COVID” patients had higher scores of CES-D (*p* = 0.01 and *p* = 0.04, respectively) and IES-R (*p* = 0.049 and *p* = 0.02, respectively), while HADS depression was higher only at T2 (*p* = 0.02). At T1, caregivers of non-survivors had higher scores of CES-D (27.6 ± 10.6 vs 15.3 ± 6.7, *p* = 0.005) and IES-R (27.7 ± 10.0 vs 17.2 ± 9.6, *p* = 0.03). We also found a significant increase in CES-D at T2 in ICU-survivors (*p* = 0.04).

**Conclusions:**

Our preliminary results showed that a video-call implementation strategy between caregivers and patients admitted to the ICU is feasible. However, this strategy did not show an improvement in terms of the risk of depression, anxiety, and PTSD among caregivers. Our pilot study remains exploratory and limited to a small sample.

**Supplementary Information:**

The online version contains supplementary material available at 10.1186/s44158-022-00067-2.

## Introduction

A fair proportion of intensive care unit (ICU) survivors may develop long lasting or even permanent disability with reduction in health-related quality of life (HRQOL); moreover, a wide spectrum of neurocognitive impairments and/or psychiatric disorders may develop after ICU discharge [1]. These physical and psychological conditions are also grouped and known as post-intensive care syndrome (PICS), which is described in up to a third of ICU survivors. Of note, the stress caused by the ICU admission triggers a burden of psychological disorders in his/her family members [2]. This condition is known as “post-intensive care syndrome-family” (PICS-F) [3, 4]. The main psychological conditions associated with both PICS and PICS-F are anxiety, depression, and post-traumatic stress disorder (PTSD) [5]. In this context, it has been shown that the presence of family members at bedside not only produces benefits for the patients and for caregivers but also enhances compliance to therapies and acceptance of sad news [6]. Conversely, one of the mechanisms by which the ICU admission could worsen the psychological outcomes of the patients and their family members is the physical detachment, which has reached the zenith during the social distancing imposed by the coronavirus disease 2019 (COVID-19) pandemic [7, 8].

According to the World Health Organization, the coronavirus pandemic has caused over 530 million infections and over 6.3 million deaths to date [9], with a high percentage of hospitalizations and ICU admissions. It is estimated that, among hospitalized COVID-19 patients, 1 in 5 requires intensive ICU admission [10, 11], and survivors may have significant impairment in HRQOL [12]. During the COVID-19 pandemic, hospital visits by relatives have been legally restricted, also for non-COVID-19 patients [13]. These restrictions have been implemented worldwide, causing in turn an inevitable separation between patients and their families [14]. Moreover, even when restrictive measures have been loosened and entrance has been gradually allowed, the use of personal protective equipment (PPE) has still blunted the contact between the family members and their loved ones.

In the impossibility of allowing in-person visits during the pandemic, we implemented video communication between patients and their loved ones, judging it as the most simple and feasible method to reduce such detachment. We hypothesized that implementation of video-calls could reduce the negative psychological effects on anxiety, depression, and PTSD in caregivers of ICU patients admitted during the pandemic.

## Methods

This prospective study was conducted at the ICU of the Policlinico “G. Rodolico” University Hospital (Catania) from 6 October 2020 to 18 February 2022. The study was approved by the local ethics committee (Registration code: 38/2022/PO). We evaluated the effects of the introduction of a video-call performed twice per week on the incidence of depression, anxiety, and PTSD in the caregivers of our ICU patients.

### Video-calls

**Fig. 1:**
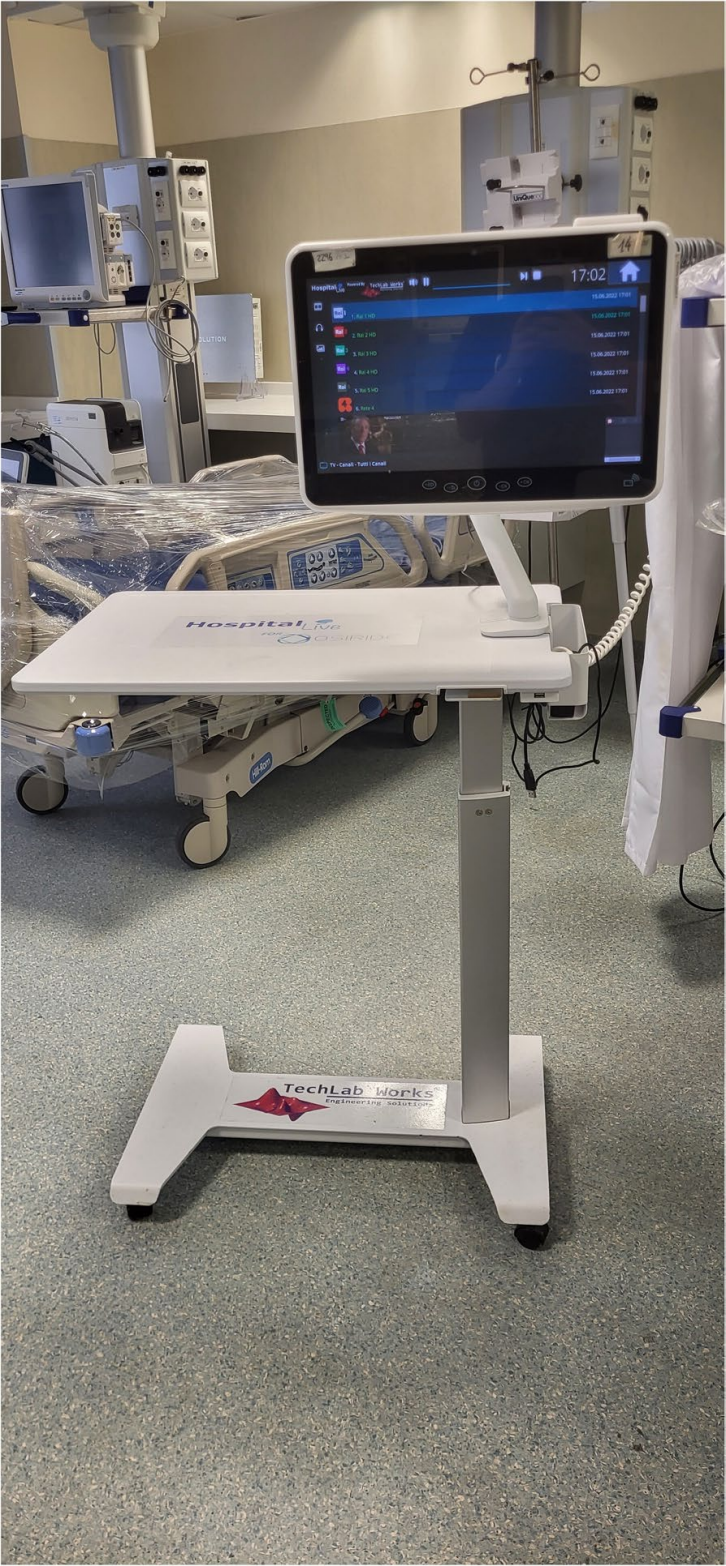
OSIRIDE video-call device

At our center, we gradually implemented an innovative international project called “OSIRIDE” promoted by our region [15] and based on the use of video terminals for improving patient comfort (Fig. 1). The availability of this device at bedspace aims at virtually reducing the distance between patient and his/her loved ones, as well as making more pleasant the hospital stay. The OSIRIDE device system allows patients to have, in very simple way and guided by a tutor, several possibilities as games, chance to read newspapers, watch movies, or listen to music; above all, OSIRIDE allows chatting and video-call with family members, which in turn can send photos, music, and videos. However, during the pandemic conditions and due to shortage of personnel and high workload, OSIRIDE terminals were used only for the purpose of video-calls, which were performed twice a week by a trained nurse of the OSIRIDE project under the supervision of doctors working in the ICU.

### Periods, groups, and criteria for the study

The project started gradually when our unit was converted to COVID-19 ICU and thus included only caregivers of COVID-19 patients admitted during the period from 06 October 2020 to 10 March 2021. However, the first video-call was performed over 1 month after conversion to COVID-19 ICU. As a subsequent reconversion of our unit to “non-COVID-19 ICU” (starting from 11 March 2021), with pandemic restriction still in place, we decided to carry on the study including caregivers of non-COVID-19 patients for whom a direct visit was not allowed or feasible. The study was concluded on the 18 February 2022 as it was disposed that family members of non-COVID-19 ICU patients could enter the hospital and ICU after antigen testing for COVID-19.

Eligibility criteria for the study were as follows:

1. ICU admission, regardless of the respiratory support and the level of consciousness
2. Expected ICU length of stay (LOS) over a week
3. Consent of the family to take part in the study
4. Family not allowed or not willing to physically visit in the ICU

If the patient died or was discharged before the third video-call, or the caregivers were allowed to enter the ICU, they were excluded from the study. Caregivers could withdraw the consent at any time during the study.

### Outcomes

Assessment of anxiety, depression, and PTSD was done using the following questionnaires validated in Italian (filled online): Impact of Event Scale [16] (Revised IES-R, Supplementary material), Center for Epidemiologic Studies Depression Scale [17] (CES-D, Supplementary material), and Hospital Anxiety and Depression Scale [18] (HADS, Supplementary material). All these questionnaires had a validated Italian version that was used in this study [19–21].

We included in the study only caregivers answering the questionnaire twice. Video-calls were performed twice per week, but questionnaires assessing the psychological outcomes were filled in two occasions. In particular, the questionnaires were completed: for the first time (T_1_) before the first video-call and for the second time (T_2_) before the third video-call (performed at least 1 week apart from the first one). The decision to set *T*_2_ at least 1 week after the first video-call was taken as the psychological questionnaires referred to the caregiver’s mood in the past 7 days. Eligible family members were contacted before the video-call in order to give their consent for the study and if in agreement they were asked to fill out the Italian version of the questionnaires which were sent in the form of a Google Sheet via message through a mobile phone or email, as preferred by the caregivers. Caregivers did not have a time limit to fill up the questionnaire, and we did not quantify this aspect.

We planned to analyze the questionnaire results dividing the population in subgroups according to the admission diagnosis and to patient’s survival. With reference to the admission diagnosis, we analyzed both the differences between different timepoints (T1 vs T2 scores) in COVID-19 or in “non-COVID” caregivers, as well as the differences at single timepoint (i.e., T1 scores in COVID-19 vs T1 scores in “non-COVID”).

## Results

During the study period, we admitted 102 COVID-19 patients and 305 non-COVID-19 patients. A vast majority of patients were excluded as their expected ICU-LOS was estimated shorter than 1 week. Other causes were lack of personnel to facilitate video-calls, technological barriers in the use of web-based questionnaires, and changes in directives regarding the physical distancing. While all ICU admissions were screened for potential inclusion in the study, due to the high workload imposed by the pandemic, we did not track the reasons for each exclusion from the study.

Overall, we included 20 caregivers (*n* = 12 from COVID-19 patients and *n* = 8 from non-COVID-19 ones) from 17 patients (11 of them admitted with COVID-19). Regarding the six non-COVID-19 patients, their admission diagnosis was as follows: 3 respiratory failure, 1 hemorrhagic shock, 1 status epilepticus, and 1 return of spontaneous circulation after cardiac arrest.

A total of 11 patients survived (*n* = 9/11 in the COVID-19 group and *n* = 2/6 in the non-COVID-19 group). Table 1 shows all the characteristics of patients and caregivers. Regarding ventilatory support in the included patients, both conditions before and after the video-calls are shown.

**Table 1:** Demographics of patients and caregivers

Patients			
	**All**	**COVID-19**	**Non-COVID-19**
**Number**	17	11	6
**Age**	64.2 ± 17.7	66.8 ± 13.1	59.3 ± 25
**Male**	14 (82.3%)	9 (81.8%)	5 (83.3%)
**Ventilation support at *T***_**1**_	6 IMV, 9 NIV, 2 HF	9 NIV, 2 HF	6 IMV
**Ventilation support at *T***_**2**_	6 IMV, 5 NIV, 6 HF	1 IMV, 5 NIV, 5 HF	5 IMV, 1 HF
**Increased respiratory support (*T***_**1**_**–*T***_**2**_**)**	1	1	0
**Decreased respiratory support (T1–T2)**	4	3	1
**Same support between *T***_**1**_** and *T***_**2**_	12	7	5
**Survivors**	11	9	2
**Caregivers**			
**Number**	20	12	8
**Age**	38.2 ± 13.7	36.7 ± 13.8	40.4 ± 14.3
**Male**	9 (45%)	6 (50%)	3 (37.5%)

*IMV*, invasive mechanical ventilation, *NIV* noninvasive mechanical ventilation, *HF* high-flow nasal cannula

In the entire study population, the average results of the questionnaires completed by the caregivers between the two video-calls showed no significant difference in terms of depression (CES-D and HADS-D), anxiety (HADS-A), and PTSD (IES-R; Table 2).

**Table 2:** Results regarding psychological tests for depression, anxiety, and post-traumatic stress disorder between video-calls in the overall cohort of caregivers. Four psychological tests were performed

Test	*T*_1_	*T*_2_	*p*-value
CES-D	19.6 ± 10	22 ± 9.6	0.17
HADS anxiety	8.7 ± 2.4	8.4 ± 3.8	0.67
HADS depression	9.5 ± 1.6	9 ± 3.9	0.59
IES-R	20.9 ± 10.8	23.1 ± 12	0.19

*CES-D* Center for Epidemiologic Studies Depression (depression), *IES-R* Impact of Event Scale-Revised, *HADS* Hospital Anxiety and Depression Scale (depression or anxiety version)

Table 3 shows the results of two subgroup analyses. The first analyzed the score change in questionnaires over time (from *T*_1_ to *T*_2_) separating COVID-19 from non-COVID-19 group: in this “timing analysis,” we found no differences between groups. Conversely, the second subgroup analysis was conducted evaluating differences in questionnaire scores between groups (“diagnosis analysis”) and demonstrated significant differences between groups. Indeed, results of CES-D and IES-R were significantly higher in the non-COVID-19 group both at *T*_1_ and *T*_2_. Moreover, at *T*_2_ we found significantly higher HADS depression scores (*p* = 0.02) and a trend toward higher HADS anxiety scores (*p* = 0.08).

**Table 3:** Subgroup analyses regarding the tests for depression, anxiety, and post-traumatic stress disorder between video-calls (“timing”) and according to the reason for intensive care admission (“diagnosis”)

Test		*T*_1_	*T*_2_	*p*-value (timing)
CES-D	**COVID-19**	15.3 ± 7.8	18.3 ± 8	*p* = 0.13
	**Non-COVID-19**	26 ± 9.8	27.4 ± 9.7	*p* = 0.69
	***p*-value (diagnosis)**	***p* = 0.01**	***p* = 0.04**	/
HADS anxiety	**COVID-19**	8.3 ± 2.2	7.2 ± 4	*p* = 0.37
	**Non-COVID-19**	9.4 ± 2.7	10.1 ± 2.8	*p* = 0.47
	***p*-value (diagnosis)**	*p* = 0.32	*p* = 0.08	/
HADS depression	**COVID-19**	9 ± 0.9	7.4 ± 3.5	*p* = 0.15
	**Non-COVID-19**	10.1 ± 2.2	11.4 ± 3.3	*p* = 0.34
	***p*-value (diagnosis)**	*p* = 0.12	***p* = 0.02**	/
IES-R	**COVID-19**	17.1 ± 9.9	18.2 ± 10	*p* = 0.45
	**Non-COVID-19**	26.6 ± 10	30.4 ± 11.5	*p* = 0.31
	***p*-value (diagnosis)**	***p* = 0.049**	***p* = 0.02**	/

*COVID-19* Coronavirus disease 2019, *CES-D* Center for Epidemiologic Studies Depression (depression), *IES-R* Impact of Event Scale-Revised, *HADS* Hospital Anxiety and Depression Scale (depression or anxiety version)

Finally, Table 4 shows the results of the subgroup analysis according to ICU survival. In particular, at T1, the caregivers of non-survivors had higher scores of CES-D (27.6 ± 10.6 vs 15.3 ± 6.7, *p* = 0.005) and IES-R (27.7 ± 10.0 vs 17.2 ± 9.6, *p* = 0.03). The other results were not significant. Comparing results of caregivers’ questionnaires over the time according to the patient’s survival, the only difference was a significant increase in CES-D at T2 in ICU survivors (*p* = 0.04).

**Table 4:** Subgroup analyses according to the results of questionnaires for depression, anxiety, and post-traumatic stress disorder between video-calls (“timing”) according to the patient’s survival in the intensive care unit (ICU)

Test		T_1_	T_2_	*p*-value (timing)
CES-D	**ICU survivors**	15.3 ± 6.7	20.2 ± 10.5	***p* = 0.04**
	**Non survivors**	27.6 ± 10.6	25.1 ± 7.4	*p* = 0.19
	***p*-value (survival)**	***p* = 0.005**	*p* = 0.29	
HADS anxiety	**ICU survivors**	8.5 ± 2.4	7.1 ± 3.9	*p* = 0.23
	**Non survivors**	8.1 ± 4.3	9.6 ± 4.6	*p* = 0.57
	***p*-value (survival)**	*p* = 0.83	*p* = 0.23	
HADS depression	**ICU survivors**	9.1 ± 1.0	8.5 ± 4.5	*p* = 0.61
	**Non survivors**	8.9 ± 4.4	8.7 ± 4.4	*p* = 0.96
	***p*-value (survival)**	*p* = 0.82	*p* = 0.93	
IES-R	**ICU survivors**	17.2 ± 9.6	19.9 ± 12.6	*p* = 0.14
	**Non survivors**	27.7 ± 10	28.9 ± 8.7	*p* = 0.74
	***p*-value (survival)**	***p* = 0.03**	*p* = 0.11	

*CES-D* Center for Epidemiologic Studies Depression (depression), *IES-R* Impact of Event Scale-Revised, *HADS* Hospital Anxiety and Depression Scale (depression or anxiety version)

## Discussion

We conducted this feasibility study to provide families and patients with greater psychological support under the constraints of a physical detachment imposed by the pandemic conditions. We used a new and innovative method from an innovative international project called “OSIRIDE” supported by our region [15]. This project is based on the availability of video terminals at bedspace in order to improve patient comfort during their hospital stay. The device enables to virtually reduce detachment of the patient from his/her family. Although “OSIRIDE” device system allows patients to have multiple advantages (games, newspapers, movies, music), the main use of this platform is for video-call with family members, and due to the challenges imposed by the pandemic workload, “OSIRIDE” terminals were used only for this purpose.

In this pilot study, we showed that implementation of video-calls is feasible in the ICU, even in the context of a pandemic with unprecedented workload. It has been shown that telephone and virtual visits became the primary communication methods in many ICUs during the pandemic [22]. Feasibility of video-calls is supported by previously published studies [23, 24], although an uneven distribution in the access to technology by the caregivers enrolled may be a concern [25]. Our single-center prospective study confirmed these results about the feasibility of the implementation of a videocall strategy between caregivers and patients. However, it should be noted that only a minority of families completed two questionnaires and were enrolled in the study.

In a recent Italian cross-sectional study, Galazzi et al. [8] showed that a video-call service to caregivers of 70 “end-of-life” ICU patients with COVID-19 was judged favorably by family members. In particular, nearly all of those who made a video-call would have done it again (27/28, 96.4%), whereas nearly all of those who did not make a call (26/28, 92.9%) wished they had.

Also, another study which conducted semistructured interviews on caregiver of ICU patients showed that 65% of family members had symptoms of depression, anxiety, or post-traumatic stress [26].

Before looking at the results of the questionnaires in caregivers of ICU patients, one should keep in mind that our study was a pilot project focusing on feasibility. Our study may help the design of future studies facilitating among others the sample size calculation. Conversely, our study lacked a control group of caregivers not performing the video-calls, and therefore could not test if video-calls are an effective strategy in reducing psychological distress. Having said that, our study did not show differences in the four psychological questionnaires filled in by caregivers of ICU patients between the two timepoints. However, by simply looking at the mean scores, one can see that their values are similar or eventually higher at *T*_2_. Moreover, the absence of improvements in the psychological scores over time (“subgroup analysis timing”) was consistent between in both subgroups of caregivers of COVID-19 and non-COVID-19 ICU patients. Considering the reduced sample size is difficult to state that the opportunity to perform video-calls given to the caregivers of ICU patients does not have positive effects. For instance, several other benefits provided by the video-calls were not evaluated in our study. Among these, we did not investigate if the video-calls were able to increase the trust of the families in the work performed by healthcare providers and in the level of care provided to the patients. Moreover, it is possible that in the absence of video-calls, the scores at *T*_2_ would have been even higher. Whether video-calls may mitigate psychological impact on caregivers due to the admission of their loved one to the ICU remains to be established.

One interesting finding of our study was the significantly higher scores of CES-D and IESR in the caregivers of non-COVID-19 patients, which was visible at both timepoints. Indeed, mean CES-D scores at *T*_1_ and *T*_2_ were 26 and 27 points respectively in non-COVID-19 caregivers, as compared to values of 15 and 18 in caregivers of COVID patients, with a mean score difference of 50% or higher. The same finding was reported looking at mean scores of the IESR questionnaires in the non-COVID-19 group (26 and 30 points at *T*_1_ and *T*_2_, respectively), as compared to the COVID-19 group (17 and 18 points, at *T*_1_ and *T*_2_, respectively). Moreover, some differences (significant or trend) were seen also for the other two HADS questionnaires at *T*_2_. The other subgroup analyses were conducted according to the patient’s survival. Interestingly, we found significantly higher CES-D and IES-R scores in caregivers of non-survivors. This may suggest a greater stress on the caregivers of patients with more severe prognosis. Nonetheless, considering the small sample size of our study, we do not feel to overinterpret such differences due to several possible confounders. Indeed, in our non-COVID-19 group, all patients were sedated and intubated, probably leading to a greater stress in their caregivers, while no one of the COVID-19 patients was intubated at *T*_1_; being awake with NIV or HFNC support, the video-call of COVID-19 patients with their caregiver(s) may have been certainly more interactive.

On reflection, our study has the value of evaluating an approach for facilitating the communications between caregivers and their loved one in a period of social restrictions. It must be noted that over half of patients who survived a period of prolonged mechanical ventilation will need significant assistance from caregivers at 1 year after ICU discharge [27]. It is estimated such unpaid labor of caregivers accounts for US $27 billion/year in Canada and US $642 billion/year in the USA [28]. Although caregivers’ assistance is likely beneficial for the patient, this burden has negative consequences for caregivers who experience an increased risk of mental health morbidity. Of note, family members during the pandemic may be particularly vulnerable as it is challenging to allow physical presence at bedspace, which seems one of the strategies protecting them against sense of grief and guilt. Indeed, it has been recommended to promote flexible ICU visitation policies for family members as a crucial step toward patient- and family-centered care [29]. Versatile visiting hours and the presence of family members have beneficial effects on delirium prevention [30–32] and stress reduction [33], both in patients and their family members. Furthermore, physical presence of caregivers allows more effective communication [34], with greater transparency and better understanding of decision-making processes from clinicians. Moreover, family members at bedspace motivate the patient, making more acceptable the discomfort associated with the burden of care. Unfortunately, in the context of COVID-19 pandemic, family members have been unable to see their loved one in the ICU, with several possible negative effects. In light of these considerations, after over 1 year of pandemic, an Italian consensus of experts suggested why and how to open ICU to visits again, despite pandemic was not over yet. The experts stressed the importance of allowing family visits even for short periods of time, especially for those that could benefit the most. Meanwhile, when visiting must be restricted or it is not feasible, additive strategies should be considered to enhance clear communication about patient comfort and to reassure family members. In this regard, video technology may allow family members to assess their loved one’s comfort level by themselves and may motivate non-sedated patients.

Our study has several limitations. As mentioned, there is lack of a control group not performing the video-calls. Therefore, the study does not answer the question whether video-calls are an effective strategy in reducing the psychological distress of caregivers of ICU patients. Rather, our study focuses on the feasibility of the implementation of the video-calls during a period of extreme workload and stress for the healthcare personnel. Furthermore, the small sample size hampers the interpretation of the findings, and therefore, our investigation should be interpreted as a feasibility study. Initiatives as the one presented in our study can be seen in a wider project aiming at reducing barriers between patients/families and healthcare providers, possibly increasing trust in the service provided and mitigating the risk of mental health problems in patients and in their caregivers after the discharge from ICU. Among these initiatives, the In:SPIRE intervention, a multidisciplinary support program implemented in Scotland, has shown to improve emotional health and reduce symptoms of insomnia in ICU survivors. The aim is to provide both ICU patients and their caregivers with support for reducing the risk of mental health issues [35, 36].

## Conclusions

Our pilot study showed the feasibility of implementing a system of video-calls for communication between ICU patients and their caregivers under the constraints of the current pandemic. Our preliminary results showed that such video-call implementation strategy did not decrease the risk of depression, anxiety, and PTSD, and our results appear similar regardless the reason for ICU admission (COVID-19 or different). Our study remains exploratory, and larger samples of caregivers are needed to characterize the benefits of implementing video-calls.

## Supplementary Information

**Additional file 1.** Survey questions.

## Data Availability

The datasets used and/or analyzed during the current study are available from the corresponding author on reasonable request.
