# Supplementary material for: Implementation of video-calls between patients admitted to intensive care unit during the COVID-19 pandemic and their families: a pilot study of psychological effects
Source: J Anesth Analg Crit Care. 2022 Aug 23;2:38. doi: 10.1186/s44158-022-00067-2 (PMC9397160; doi:10.1186/s44158-022-00067-2)

Supplementary Material

Survey questions

1. Nome e Cognome dell'Intervistato
2. Età dell'intervistato
3. Sesso dell'intervistato
4. Grado di istruzione dell'intervistato
5. Cognome e Nome del parente ricoverato?
6. Legame con il paziente?
7. Ha già visto tramite videochiamata il suo parente dal momento del ricovero in terapia intensiva?
8. Mi sentivo annoiato da cose che normalmente non mi annoiano?
9. Non avevo voglia di mangiare: avevo poco appetito?
10. Sentivo che non riuscivo a scrollarmi di dosso la tristezza nonostante l'aiuto e il supporto della mia famiglia o degli amici?
11. Sentivo di essere bravo quanto le altre persone?
12. Avevo difficoltà a mantenere la concentrazione su ciò che stavo facendo?
13. Mi sentivo depresso?
14. Sentivo che ogni cosa che facevo richiedeva sforzo?
15. Mi sentivo pieno di speranza per il futuro?
16. Sentivo che la mia vita era stata un fallimento?
17. Mi sentivo spaventato?
18. Il mio sonno era senza riposo?
19. Mi sentivo felice?
20. Parlavo meno del solito?
21. Mi sentivo solo?
22. Le persone non erano amichevoli con me?
23. Mi godevo la vita?
24. Avevo crisi di pianto?
25. Mi sentivo triste?
26. Sentivo che le persone non mi apprezzavano?
27. Non riuscivo ad andare avanti?
28. Ogni ricordo mi riportava a quell'evento?
29. Avevo difficoltà a rimanere addormentato?
30. Altre cose mi facevano pensare a quell'evento?
31. Mi sentivo irritabile e arrabbiato/a?
32. Cercavo di non lasciarmi sopraffare quando pensavo o ricordavo quell'evento?
33. Ci pensavo anche quando non volevo?
34. Avevo la sensazione che non fosse mai accaduto o non fosse vero?
35. Cercavo di allontanarmi da ciò che me lo faceva ricordare?
36. Immagini dell'evento mi riaffioravano nella mia mente?
37. Ero teso e facilmente impressionabile?
38. Provavo a non pensarci?
39. Ero consapevole di essere ancora provato/a dall'evento, ma non volevo occuparmene?
40. Le mie emozioni per l'evento erano come intorpidite?
41. Mi trovavo ad agire o a sentire come se fossi tornato/a indietro a quel momento?
42. Avevo difficoltà a prendere sonno?
43. Avevo ondate di forti emozioni per l'evento?
44. Mi sento agitato e teso?
45. Provo un sentimento di apprensione come se dovesse succedere qualcosa di tremendo?
46. Mi passano per la mente pensieri preoccupanti?
47. Riesco a sedermi e rilassarmi?
48. Provo un sentimento simile alla paura, come un senso di tensione allo stomaco?
49. Mi sento irrequieto ed incapace di stare fermo?
50. Provo delle sensazioni improvvise di panico?
51. Le cose che un tempo mi piacevano, ora mi piacciono?
52. Riesco a ridere e vedere il lato divertente delle cose?
53. Mi sento di buon umore?
54. Mi sento rallentato?
55. Non curo più il mio aspetto fisico?
56. Pregusto con piacere le cose?
57. Provo piacere leggendo un libero, oppure seguendo una trasmissione alla radio o alla TV:
58. Quante volte ha effettuato la videochiamata con il suo parente?
59. In una scala da 1 a 10 come valuta il servizio di Rianimazione e Terapia Intensiva dell'Ospedale Policlinico?


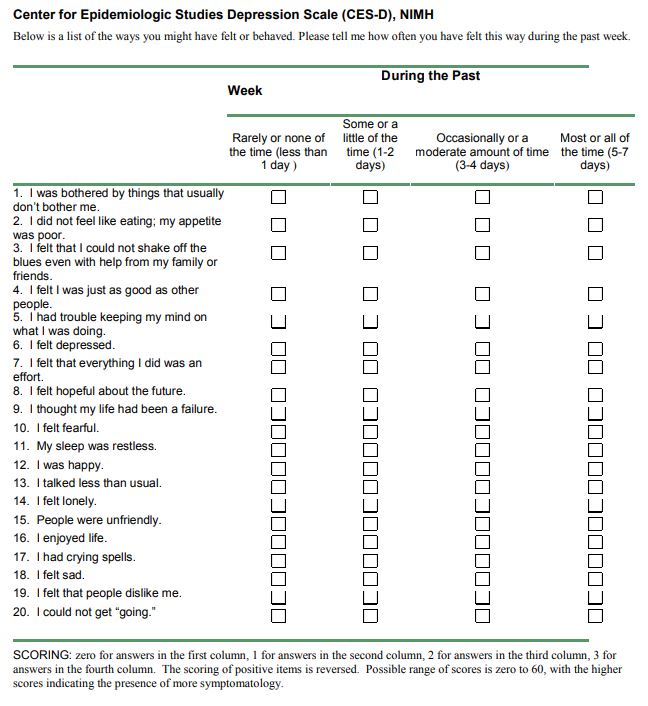


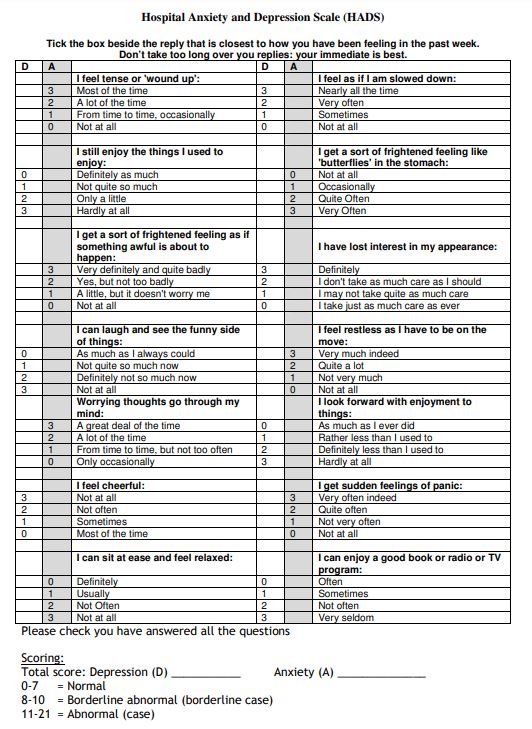

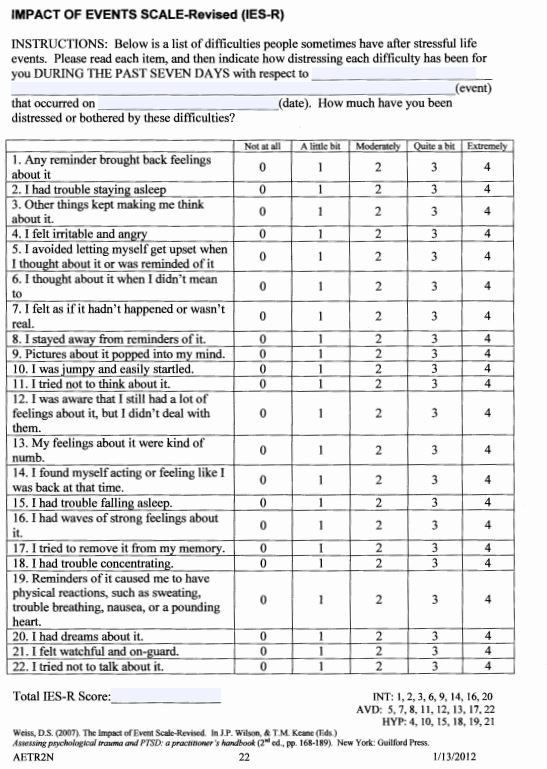

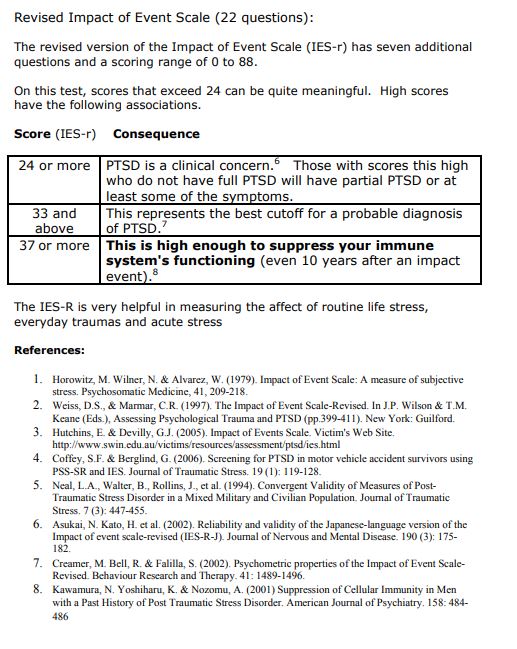

Supplement: Supplementary file 1 — Additional file 1. Survey questions. [file 44158_2022_67_MOESM1_ESM.docx]
